# The COVID-19 may help enlightening how emotional food is

**DOI:** 10.1038/s41538-020-00071-2

**Published:** 2020-08-12

**Authors:** Géraldine Coppin

**Affiliations:** 1 Department of Psychology, Swiss Distance University Institute, Brig, Switzerland; 2 Swiss Center for Affective Sciences, University of Geneva, Geneva, Switzerland

**Keywords:** Human behaviour, Science, technology and society

## Abstract

Olfactory and gustatory stimuli can elicit potent emotional responses and are essential in food perception. Yet, main theories of emotion often under-represent them, and our understanding of affective phenomena relies mostly on experimental studies conducted on visual and auditory stimuli. Although evidence is still accumulating today, recent findings suggest that the COVID-19 is associated with a loss in olfaction and/or taste. Here, I discuss how this unprecedented and uncommon spread of the loss of olfaction and/or taste worldwide may enlighten how emotional both these senses are and how much they influence food perception.

## Introduction

Humans have a much better sense of smell than often believed (see ref. ^1^). Humans are able to smell billions of different odors^2^ and they can follow a scent trail in a field like dogs^3^. Olfactory and gustatory stimuli elicit potent emotional responses (e.g. refs. ^4,5^), sometimes even more potent than visual or auditory stimuli (e.g. ref. ^6^). Yet, main theories of emotion often under-consider them. For instance, odor-evoked emotions do not fit into basic emotions categories (see ref. ^7^). In terms of empirical data, one can make a similar observation as what prevails with theories of emotion. Although a small community of researchers investigates the bidirectional links between olfaction, taste, flavor, food and affective phenomena (e.g. ref. ^8^), our understanding of emotions relies mostly on experimental studies conducted on visual and auditory stimuli. Could one hope the affective sciences literature becomes more inclusive of other sensory modalities in the near future?

Although evidence is still accumulating at this point, it has been reported in more than 4,000 participants that the COVID-19 is associated with a reduction or loss of smell and/or taste^9^. Several other reports also came to the same conclusion (e.g. refs. ^10–15^). Regarding the sense of smell, this means that the perception of olfactory compounds from the outside world (i.e., orthonasal olfaction; e.g., smelling a rose) and in the mouth through food and drinks (i.e.e, retronasal olfaction; e.g., perceiving a strawberry flavor) are both altered. This perception could be diminished (hyposmia) or lost (anosmia). Consequently, what only hyposmic (5-8% of the population)/anosmic (around 1% of the population, see ref. ^16^) individuals usually get to experience, a large fraction of the planet may experience transitorily. The contrast between the fraction of people usually afflicted by these conditions and the potentially enormous number of individuals afflicted in the context of the COVID-19 sanitary crisis is even more striking when considering the sense of taste, i.e. the perception of sweet, salty, bitter, sour and umami. Dysgeusia, i.e. a reduction of taste and agueusia. i.e. the loss of taste are much rarer than hyposmia/anosmia—only 5% of patients consulting in smell or taste clinics have a taste impairment^17^. This unprecedented spread of olfactory and gustatory alterations in the population may bring a never experienced awareness of how important these sensory modalities actually are for one’s psychological functioning, including one’s emotions, especially emotions elicited by food.

Several functions of olfaction are directly connected to emotions (see ref. ^18^). A healthy sense of smell notably allows the avoidance of fear- and disgust-related environmental hazards. Anosmia comes with the fear of not being able to avoid non-microbial environmental hazards, such as gas leaks or fires^19^. But microbial hazards are also an issue: vomit, feces or putrefaction odors could elicit altered disgust reactions and potentially inappropriate behaviors without a functioning sense of smell. Moreover, the difficulty to detect and be disgusted by spoiled food could lead to the unfortunate ingestion of unsuitable food items. But even when considering perfectly edible food, many anosmic individuals also experience food-related issues. As retronasal olfaction is key in the perception of flavors (i.e., the “*perception that includes gustatory, oral-somatosensory, and retronasal olfactory signals that arise from the mouth as foods and beverages are consumed*”, see ref. ^20^, page 540), a drastically reduced hedonic value of food is common in anosmia. For instance, while eating a strawberry, an anosmic individual may perceive it as sweet and/or sour (thanks to gustatory input) but the strawberry flavor will no longer exist (due to the lack of olfactory input). Socially, anosmic individuals also report several negative outcomes: individuals can be insecure about their own body odors; feel isolated, frustrated, angry, etc. as their handicap is rarely understood (contrary to blindness or deafness); feel out of place in several social settings, such as meals, where they cannot relate. As increasing evidence suggests that human body odors convey information about emotions (for a review, see ref. ^21^), anosmic individuals may also lack crucial information about others’ emotions. Moreover, the loss of smell leads to the inability to enjoy every day smells such as cooking smells or the Spring ones, inability often associated with anhedonia. Ironically, the COVID-19 hit most countries during the Spring 2020, which could have made this aspect particularly salient. Finally, mood changes and symptoms of depression are also common in anosmic individuals (e.g ref. ^22^). Consequently, losing one’s sense of smell and/or taste comes with a high price emotion-wise and food-related behaviors are particularly hit.

Besides one’s sense of smell and/or taste, the multi-modal nature of flavors invites further consideration of how other receptors in the oral cavity, such as the ones responding to tactile, temperature and pain, are affected by the COVID-19 as well. Evidence is still sparse today but an association between COVID-19 and reported chemesthesis (responsible for burning, cooling, and tingling sensations) alterations has been reported (e.g. ref. ^9^). More generally, we are only starting to discover how the COVID-19 may impact sensory inputs related to food intake and emotions.

It is also worth nothing that the COVID-19 is not the only disease associated with a reduction or loss of smell and/or taste: epilepsia, multiple sclerosis, Parkinson’s disease, Alzheimer’s disease, cancer, etc. (see e.g. ref. ^12^) are also associated with olfactory and gustatory dysfunctions. Cancer is particularly telling regarding the importance of smell and taste for food intake: malnutrition is highly common in cancer patients, and smell and taste changes could at least partly contribute to this malnutrition, as shown by prevalence (e.g. ref. ^23^) and training (e.g. ref. ^24^) studies. In the future, attempts to tease apart and compare effects of different diseases on smell and/or taste may be particularly informative. For instance, the Global Consortium for Chemosensory Research, a group of international researchers focusing on smell and taste, is currently trying to tease apart the effects of different respiratory troubles (e.g., cold, influenza) from the COVID-19’s effects on smell and/or taste.

If the affective science literature was to include more accounts of odor- and gustatory-evoked emotions, how could it proceed? First, one would need to consider these sensory modalities, as well as touch, when revising or elaborating a theory of emotion. Vision and audition are obviously essential sensory modalities, but excluding the other ones, or multimodal stimuli, is hardly warranted. For instance, cross modal interactions between olfaction (e.g. ref. ^25^) or taste (e.g. ref. ^26^) and tactile inputs have shown to impact flavor and food perception and evaluations. More generally, multimodal stimuli appear essential to study, as emotional content of stimuli often come from several sensory modalities. Second, even though smells and tastes do come with practical constraints, their use in experimental settings could be particularly helpful in some sub-topics of science (e.g., the link between memory and emotion). These practical constraints may be worrying at first. For instance, smells and tastes need to be stored in specific conditions (e.g., temperature-wise) and their stability over the course of an experiment need to be regularly assessed. However, these contraints are easily manageable when properly trained and equipped. Devices such as olfactometers and gustometers allow the precise delivery of odors and liquids, respectively, and can be adapted to many experimental requirements. Several studies have successfully use odors and tastes in worldwide settings (e.g. ref. ^27^) or in unease contexts such as functional Magnetic Resonance Imaging (fMRI; e.g. refs. ^28,29^). It is to hope the COVID-19 sanitary crisis inspires more research on the link between olfaction, taste, flavor, food and emotions. In the long term, this could lead to theories of emotion accounting for all sensory modalities as well as their interactions, which would be a real advancement for food science.
